# Supplementary material for: A VEL3 histone deacetylase complex establishes a maternal epigenetic state controlling progeny seed dormancy
Source: Nat Commun. 2023 Apr 19;14:2220. doi: 10.1038/s41467-023-37805-1 (PMC10113200; doi:10.1038/s41467-023-37805-1)

Supplementary Figures for:

A VEL3 histone deacetylase complex establishes a maternal epigenetic state controlling progeny seed dormancy

Xiaochao Chen[1], Dana R. MacGregor[1,2], Francesca L. Stefanato[1], Naichao Zhang[1,3], Thiago Barros-Galvão[1] and Steven Penfield[1]*

Supplementary Fig. 1 Seed dormancy testing of loss of function mutants for candidate genes identified by genome-wide association.
Seeds were set at either 22°C or 16°C in order to identify mutants with more or less dormancy than Col-0 WT. Data represents minimum and maximum values as well we 25th, 50th and 75th quartiles of five biological replicates per genotype.

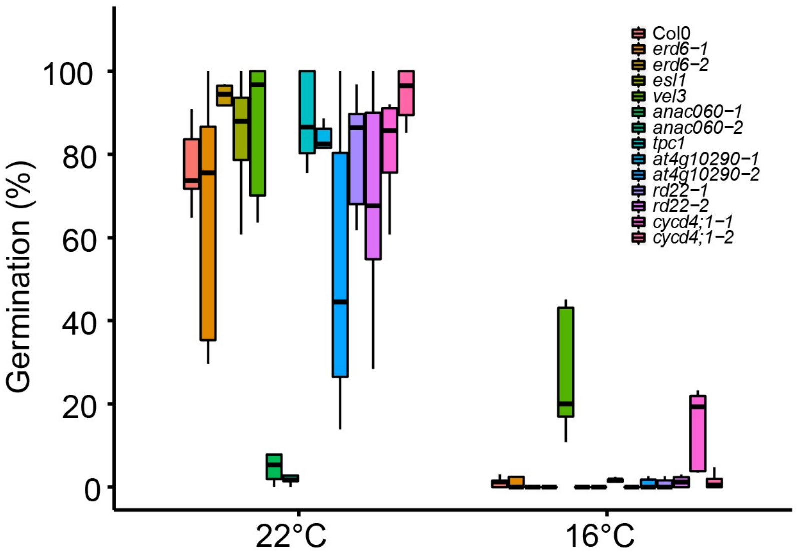

Supplementary Fig. 2 Analysis of *phyB* and *VEL2*/*VEL3* gene variation and its assocaition with seed dormancy.

(**a**) Manhattan plot derived from germination data using seeds set at 16°C and dark stratified at 16°C prior to germination (16 S16) and detail of the underlying locus. (**b**) FastPhase analysis of major haplotypes and linkage disequilibrium between the *phyB* gene and *VEL2*/*VEL3*. (**c**) Table of P-values for assocaition of *phyB* and *VEL* haplotypes with germination frequency in the 6 treatments by Generalised linear model, showing that the contributions of haplotype at both loci cannot be ruled out. (**d**) Analysis of germination frequency of accessions with the indicated hapltoypes, showing that the largest difference is between lines carrying *phyB* HAP1 and those carrying *phyB* HAP2 or 3. Lines with *phyB* HAP2 and *VEL2*/*3* HAP1 have high germination but are represented by only 4 accessions. (**e**) The maternal allele of *phyB* is most important for germination control, as shown by the germination frequencies of reciprocal crosses. Data represents minimum and maximum values as well we 25th, 50th and 75th quartiles of seven to ten biological replicates per genotype in **d-e**.

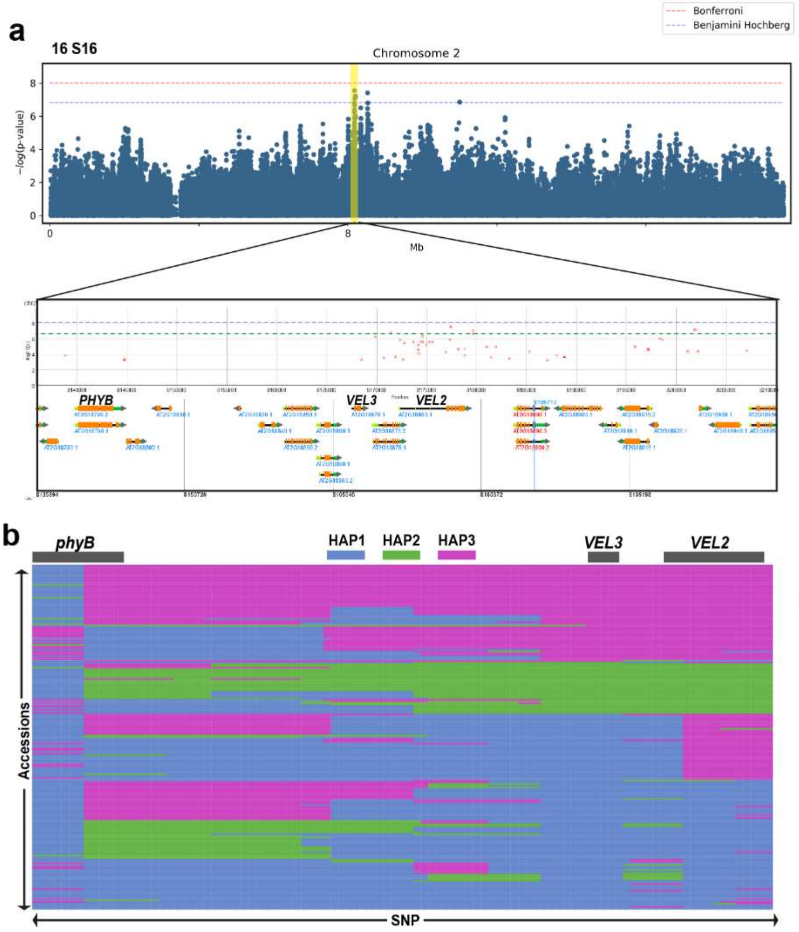

c

Treatments	phyB		VEL		phyB*VEL
	1st effect	2nd effect	1st effect	2nd effect	
22 NS	0.002	0.048	0.003	0.031	0.258
22 S16	0.058	0.057	0.060	0.055	0.313
22 S4	0.255	0.039	0.083	0.107	0.527
16 NS	0.001	0.470	0.036	0.019	0.026
16 S16	0.000	0.003	0.000	0.028	0.004
16 S4	0.329	0.034	0.014	0.885	0.028

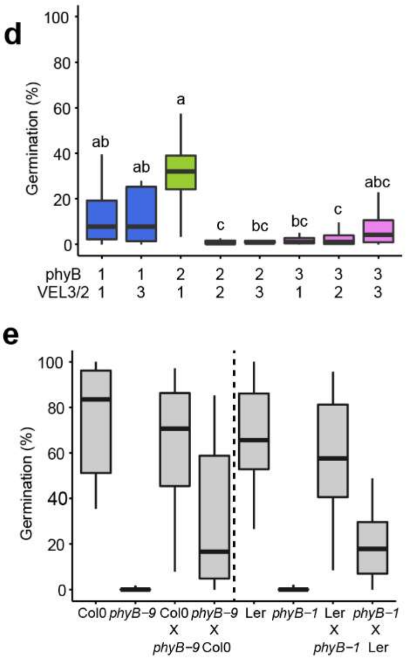

Supplementary Fig. 3 The role of VEL3 in the control of seed development.
(**a**) *vel3-2* shows higher germination than WS when set at 22°C. Data derived from 5 biological replicate seed batches per genotype. Boxplots indicate minimum and maximum values as well as 25th, 50th and 75th quartiles. (**b, c**) *vel3-2* exhibits a recessive incompletely penetrant seed abortion phenotype as seen in *vel3-1*. In panel C Total number of seeds analysed for each genotype are shown. (**d**) Time-series qRT-PCR data of the endosperm-mark gene *AGL62* in WT and *vel3-1* developing seeds, suggesting that endosperm development is delayed in *vel3-1*. Data shown are mean +/- SE of three biological replicates for each line. (**e**) Endosperm cellularization images between Col0 and *vel3-1* at heart stage. The experiments were repeated twice. (**f**) Y2H suggests that MEA shows autoactivation, and no interaction was detected between VEL3 and HD2C or HDA19. We also couldn't detect any interaction between the TAIR10-annotated VEL3 and FIS-PRC2 components.

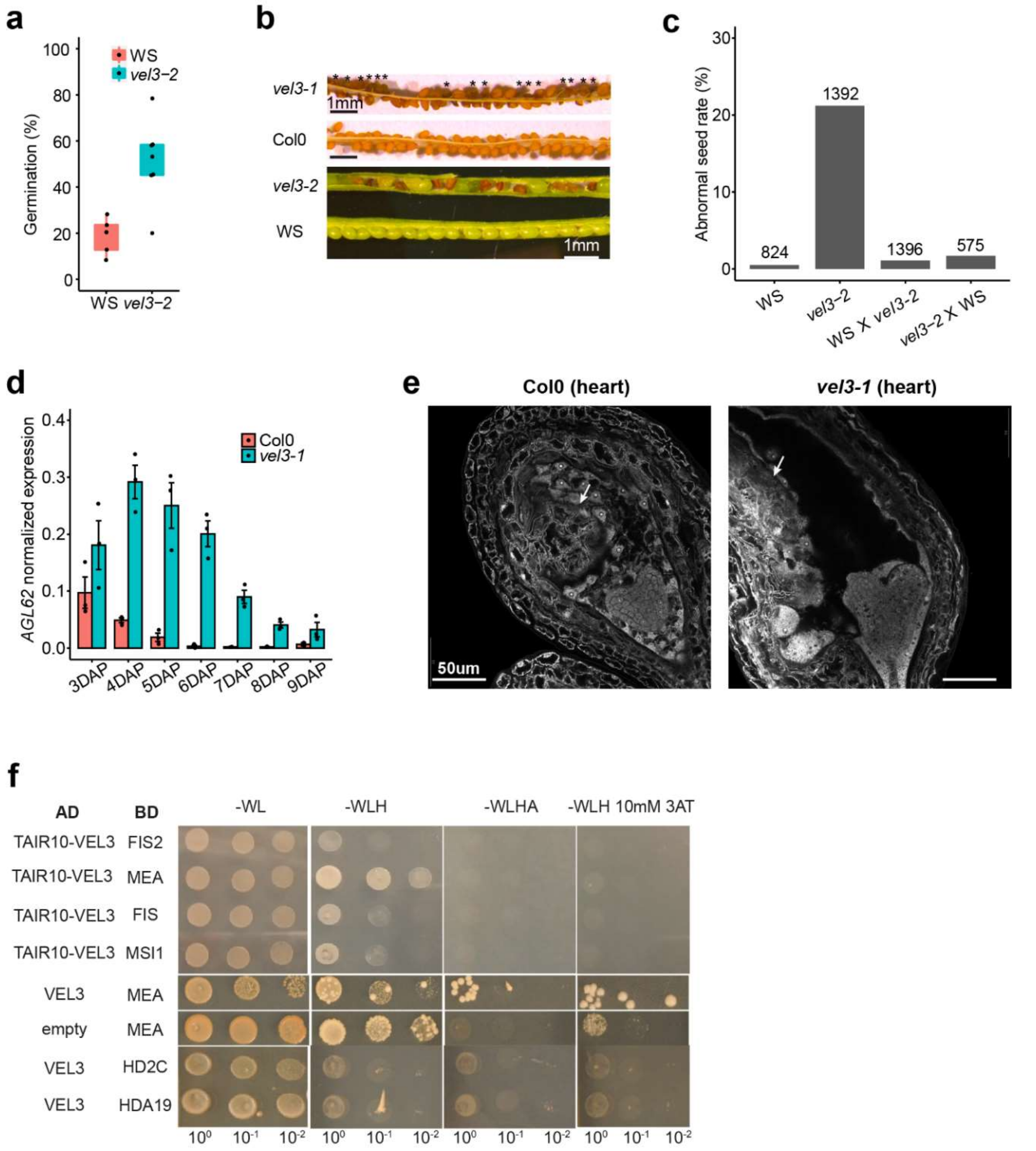

Supplementary Fig. 4 Confirmation of the exon-intron structure of the *VEL3* gene.
(**a**) Structure of the TAIR10-annotated putative cDNA (GenBank NM_127443.2), and the alternative complementing cDNA designated here *VEL3.1* (GenBank OAP08663.1). (**b**) PCR and RT-PCR from genomic DNA and cDNA revealing a *VEL3* gene product of around 800bp. The experiments were three times. (**c**) Complementation with a synthesised *VEL3.2* gene product is only partially successful, with a substantial abnormal seed rate remaining in the putative complementing lines. N=10 biological replicates. (**d**) Analysis of two lines by QPCR show that while *VEL3.2-GFP* is highly expressed re-regulation of *PHE1*, *AGL62* and other genes in the endosperm persists. Data shown are mean +/- SE of three biological replicates for each line. (**e**) the high germination of the *vel3-1* mutant is complemented by the genomic copy of *VEL3.1* in four independent lines, or in two lines expressing *VEL3.1-GFP* under the *VEL3* promoter (hereafter known as *pVEL3:VEL3*-GFP). (**f**) QPCR in developing seeds shows that the high expression of *PHE1*, *AGL62*, *CPS1* and *GA3OX4* and *GA20OX5* observed in *vel3-1* is absent in complementing *VEL3.1*-expresssing lines. Data represents mean +/- SE of 3 biological replicates per genotype. Boxplots indicate minimum and maximum values as well as 25th, 50th and 75th quartiles in **c-e**.

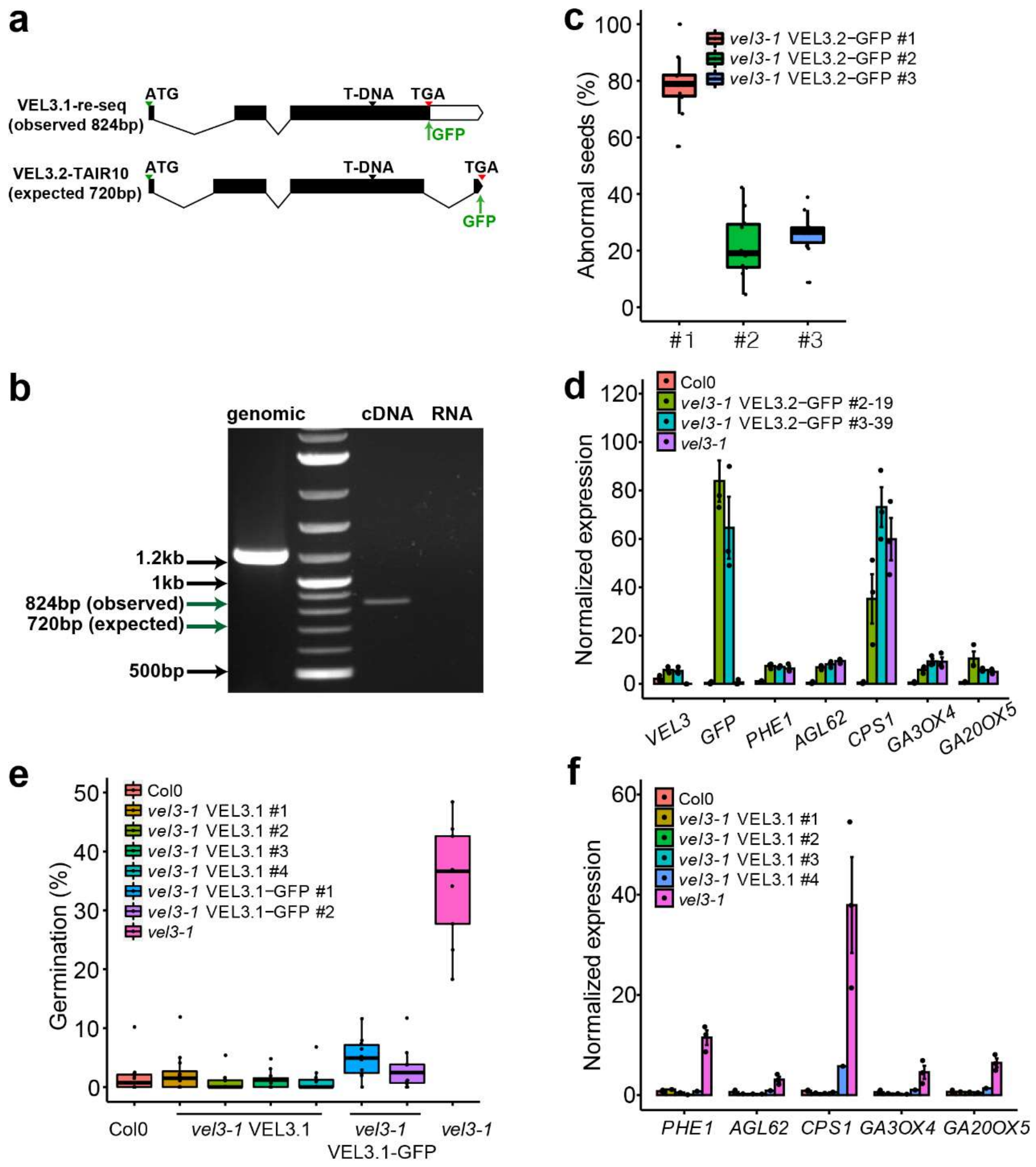

Supplementary Fig. 5 VEL3 affects H3K27me3-marked heterochromatin independently of the presence of H3K9me2 or the presence of REF6 binding sites.
Analysis of overlapping expression of genes requiring VEL3 for H3K27me3 in the mature endosperm (pink) with those marked by H3K27me3 alone (green) (**a**) of those marked by both H3K27me3 and H3K9me2 (**b**) or H3K9me2 alone (**c**). (**d**) Analysis of differentially expressed genes in *vel3-1* vs Col-0 globular stage seeds show that genes marked with H3K27me3 alone or both H3K27me3 and H3K9me2 are mis-regulated in *vel3-1* mutants. (**e**) Frequency of REF6 binding sites in genes marked by single H3K9me2, H3K27me3 and both marks in genes whose expression is up-regulated in *vel3-1* mutants. H3K27me3 and H3K9me2 data is from Moreno-Romero *et al.,* (2016). Significant overlaps were calculated by GeneOverlap in R. p values were determined using two-sided Fisher's exact test in **a-d**.

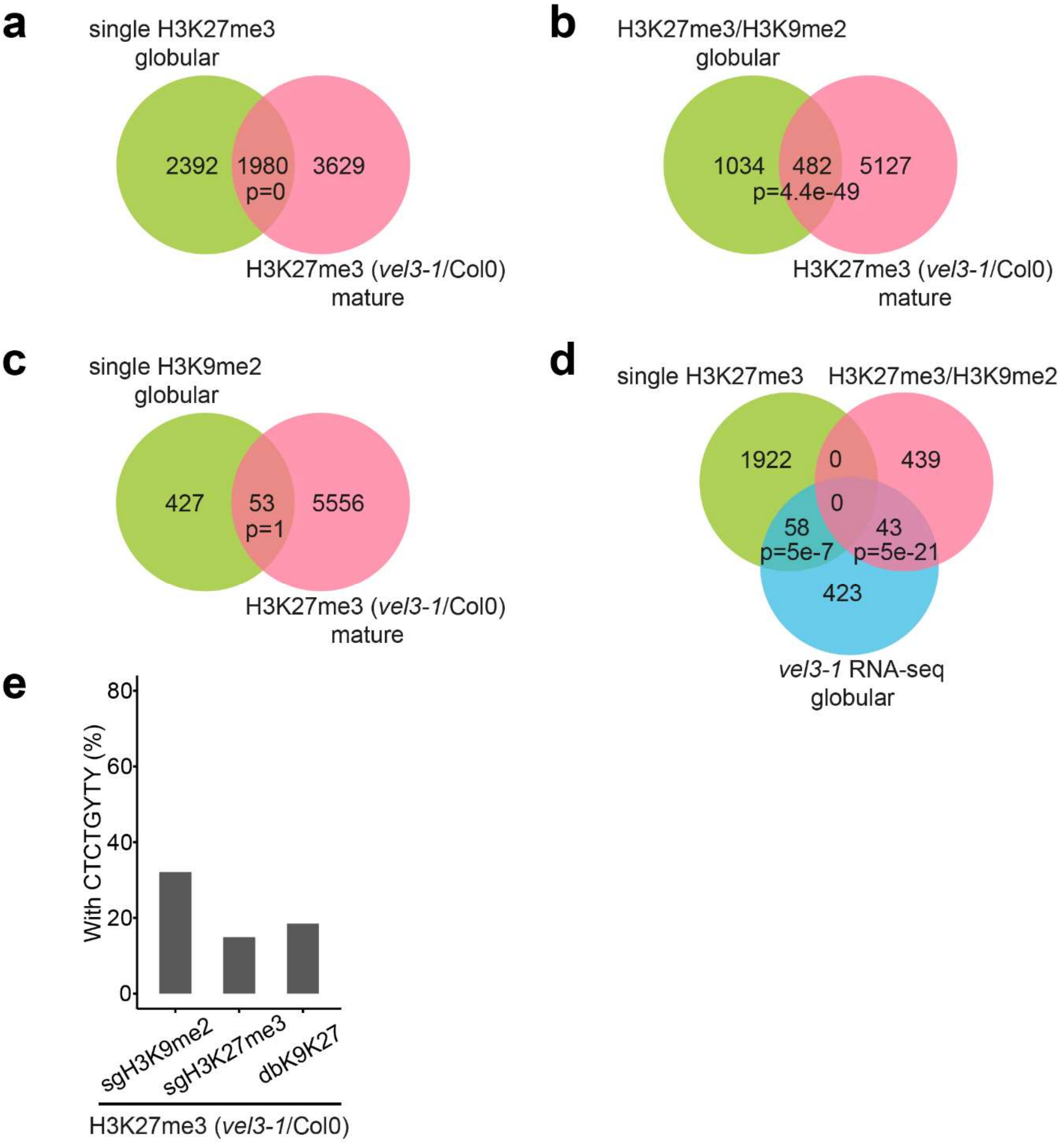

Supplementary Fig. 6 Comparison of gene expression changes in *vel3-1* vs Col-0 and those previously shown to involve loss of H3K27me3 during pathogen effector responses leading to programmed cell death.
(**a**) GO analysis of genes common between vel3-1 upregulation in the mature endosperm and those induced by the *P. syringae* effector ASvrRpt2. (**b**) GO analysis of genes common between *vel3-1* upregulation in the mature endosperm and those induced by the *P. parasitica* effector NPP1. Effector data from Tomastikova et al (2021). (**c**) GO analysis of the overlap in gene expression between *vel3-1* up-regulated genes and *ref6-1* down-regulated genes reveals a similar gene function set. (**d**) Overlaps between *ref6-1* and *vel3-1* mis-regulated genes. (**e, f**) loss of WRKY75 or NAC046 does not affect seed dormancy or germination. Seeds were set at 22°C and represent the mean +/-SE of 8 biological replicates per genotype. p values are derived from the one-sided Fisher's exact test in **a-c**.

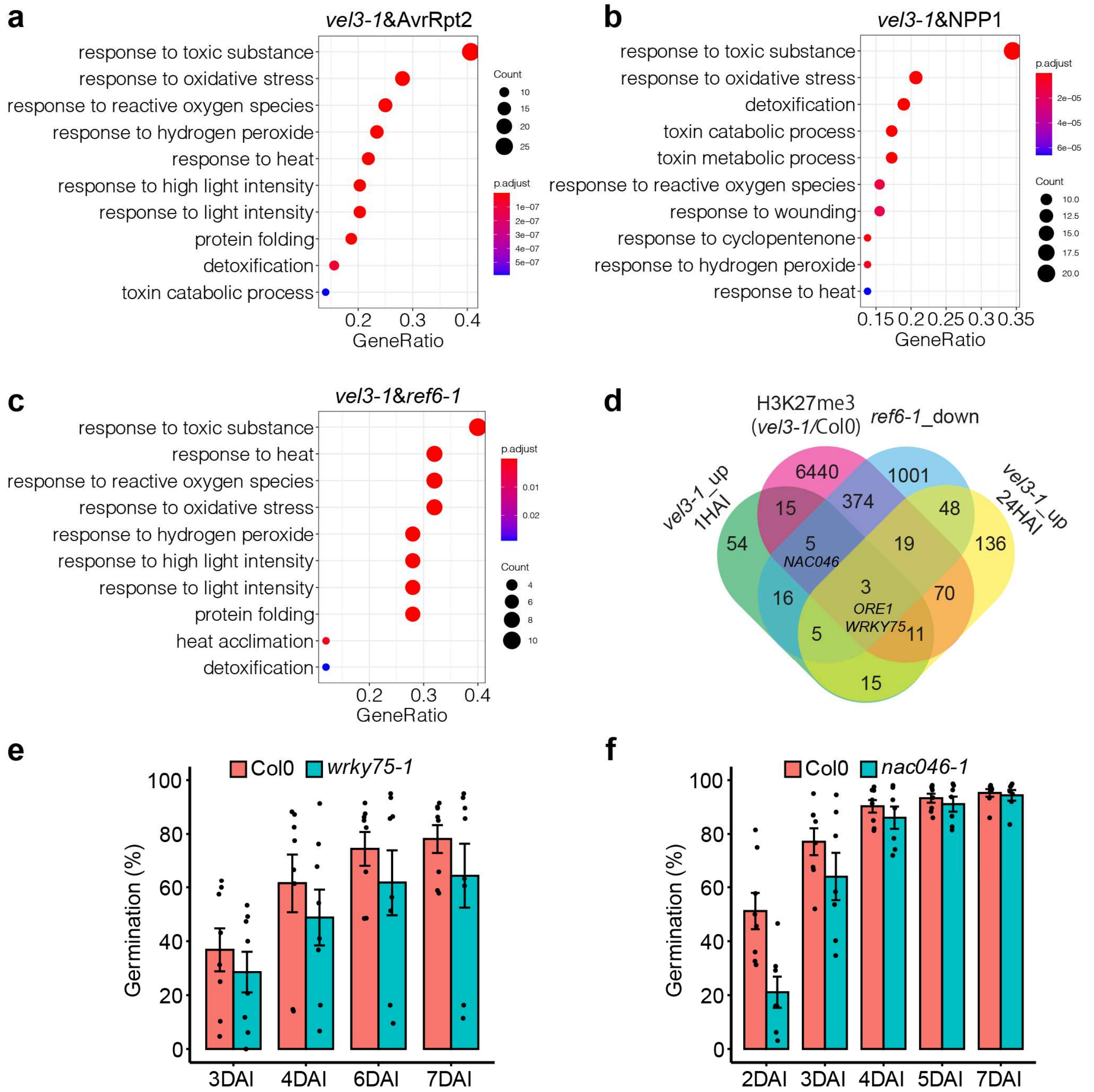

Supplementary Fig. 7 VEL3 is required for normal Histone 3 acetylation at *ORE1*, *WRKY75* and *NAC046* in the endosperm.
Chromatin immunoprecipitation using antibodies against H3Ac in mature endosperm tissues 1 hour after imbibition shows a widespread increase in H3 acetylation at the three loci. Data represent mean +/- SE of 3 biological replicates per genotype.

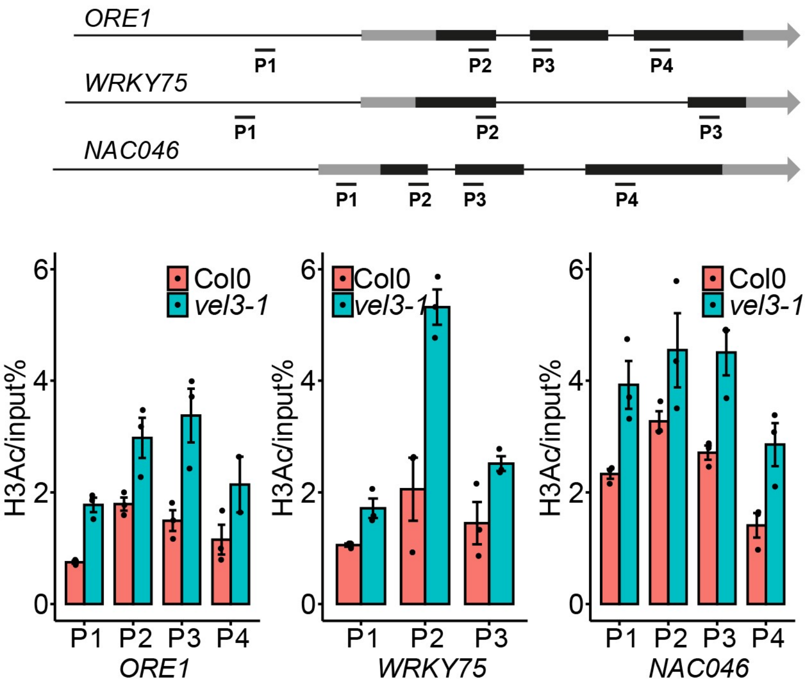

Supplement: Supplementary file 1 — Supplementary Figures [file 41467_2023_37805_MOESM1_ESM.pdf]
