# Supplementary material for: A VEL3 histone deacetylase complex establishes a maternal epigenetic state controlling progeny seed dormancy
Source: Nat Commun. 2023 Apr 19;14:2220. doi: 10.1038/s41467-023-37805-1 (PMC10113200; doi:10.1038/s41467-023-37805-1)
Supplement: Supplementary file 3 — Description of Additional Supplementary Files [file 41467_2023_37805_MOESM3_ESM.pdf]

Description of Additional Supplementary Files:

Supplementary Data 1. Seed germination of A. thaliana accessions.

Supplementary Data 2. phyB and VEL3 haplotypes of A. thaliana accessions.

Supplementary Data 3. Summary of sequencing statistics and gene lists.

Supplementary Data 4. Summary of IP-MS results.

Supplementary Data 5. Primers used in this study.
